# Genome Sequencing Unveils a Novel *Sea* Enterotoxin-Carrying PVL Phage in *Staphylococcus aureus* ST772 from India

**DOI:** 10.1371/journal.pone.0060013

**Published:** 2013-03-27

**Authors:** Sushma Prabhakara, Supriya Khedkar, Srikanth Mairpady Shambat, Rajalakshmi Srinivasan, Atanu Basu, Anna Norrby-Teglund, Aswin Sai Narain Seshasayee, Gayathri Arakere

**Affiliations:** 1 Society for Innovation and Development, Indian Institute of Science, Bengaluru, India; 2 National Centre for Biological Sciences, TIFR, GKVK, Bengaluru, India; 3 National Institute of Virology, Pune, India; 4 Karolinska Institute, Center for Infectious Medicine F59, Karolinska University Hospital, Huddinge, Stockholm, Sweden; National Institutes of Health, United States of America

## Abstract

*Staphylococcus aureus* is a major human pathogen, first recognized as a leading cause of hospital-acquired infections. Community-associated *S. aureus* (CA-SA) pose a greater threat due to increase in severity of infection and disease among children and healthy adults. CA-SA strains in India are genetically diverse, among which is the sequence type (ST) 772, which has now spread to Australia, Europe and Japan. Towards understanding the genetic characteristics of ST772, we obtained draft genome sequences of five relevant clinical isolates and studied the properties of their PVL-carrying prophages, whose presence is a defining hallmark of CA-SA. We show that this is a novel prophage, which carries the structural genes of the *hlb*-carrying prophage and includes the *sea* enterotoxin. This architecture probably emerged early within the ST772 lineage, at least in India. The *sea* gene, unique to ST772 PVL, despite having promoter sequence characteristics typical of low expression, appears to be highly expressed during early phase of growth in laboratory conditions. We speculate that this might be a consequence of its novel sequence context. The crippled nature of the *hlb*-converting prophage in ST772 suggests that widespread mobility of the *sea* enterotoxin might be a selective force behind its ‘transfer’ to the PVL prophage. Wild type ST772 strains induced strong proliferative responses as well as high cytotoxic activity against neutrophils, likely mediated by superantigen SEA and the PVL toxin respectively. Both proliferation and cytotoxicity were markedly reduced in a cured ST772 strain indicating the impact of the phage on virulence. The presence of SEA alongside the genes for the immune system-modulating PVL toxin may contribute to the success and virulence of ST772.

## Introduction

*Staphylococcus aureus* (SA) is an important bacterial pathogen of humans. It has been a major causative agent of hospital-acquired (HA) bacterial infections. It is also known for its ability to be resistant to multiple antibiotics, methicillin-resistant *S. aureus* (MRSA) being an important example [1]. Recently however, various isolates of community-associated (CA) *S. aureus* have been reported, thus widely extending the reservoirs of *S. aureus* infections. CA*-*SA strains (CA-MRSA in particular) lead to a range of simple to life-threatening skin and soft-tissue infections (SSTI) and severe abscesses, sepsis, and necrotizing pneumonia [2]. Virulence and antibiotic resistance – the two important characteristics of *S. aureus* – are generally associated with various mobile genetic elements (MGE), including several prophages and the Staphylococcal Cassette Chromosome *mec (SCCmec),* which carries determinants of antibiotic resistance [3] along with other chromosomal regulators of virulence like the *agr* system. Early comparative studies showed that among prophages, one that carries the Panton-Valentine Leukocidin (PVL) toxin is a particular hallmark of CA-SA strains [4], [5].

The PVL-carrying prophages are temperate bacteriophages belonging to the family *Siphoviridae,* which are ds-DNA phages with long non-contractile tail. These are sfi21-like *cos*-site phages and belong to one of three groups based on capsid morphology: group 1 (φPVL, φ108PVL), group 2 (φSa2958, φSa2MW, φSLT, φSa2USA) or group 3 (φ7247, φ5967, φM013). The PVL toxin itself is a bi-component, hetero-oligomeric, pore-forming cytolytic toxin comprising LukF-PV (34 KDa) and LukS-PV (33 KDa) [6]–[9]. The role of this toxin in virulence is controversial. For example, studies in rabbit infection models have suggested that PVL, though being a contributing player, could not be a major factor driving CA-MRSA infections [10], [11]. Nevertheless, it has been shown that PVL lyses human white blood cells. However, its effects are concentration dependent. At sublytic concentrations, PVL enhances innate host immunity without cell damage [12], [13]. A recent study has further demonstrated that PVL is the major trigger for IL-1β release and inflammasome activation from human macrophages [14].

The best-studied CA-MRSA strains are from the USA and are named USA300, which is highly clonal and PVL-positive. Nearly all (97%) MRSA skin infections in patients reporting emergencies in the USA are due to USA300 [15]. However, in contrast, CA-MRSA isolates from India are genetically diverse and nearly three-fourths are PVL-positive. These belong to two ST types: ST22 and ST772 [16]–[18]. While ST22 originated in England, ST772 is present mainly in India, Bangladesh and Malaysia, and has been called the Bengal Bay clone by some investigators [16], [19], [20]. But in the last couple of years, the latter has been reported from England, Ireland and Japan [21]–[23]. A recent German study, of *S. aureus* in individuals who had returned from travel to the tropics and the sub-tropics, showed that complex SSTIs were associated with PVL-positive *S. aureus* and that ST772 was predominant in individuals returning from Asia [24]. Despite this knowledge, these strains remain poorly characterized at the genetic and the molecular level.

Here we present the draft genome sequence of several ST772 isolates collected from different clinical settings, and discuss the sequence and structure of their PVL-carrying phages (with the generic name φIND772PVL) [25]. The phage sequences from the five different ST772 isolates are identical. The φIND772PVL is similar to the PVL phage from ST59 isolates (φ7247PVL and φ5967PVL for example) in that they are both mosaics including aspects of the *hlb*-converting phage. However, the φIND772PVL is unique in carrying the staphylococcal enterotoxin (*sea*) gene. We suggest that the *sea*-carrying PVL phage emerged early on within the ST772 lineage in India, on the basis of the absolute presence of the *sea* within the PVL-carrying prophage in every ST772 isolate in our collection.

## Results

### PVL-carrying Phages in *S. aureus* ST772

**Figure 1:**
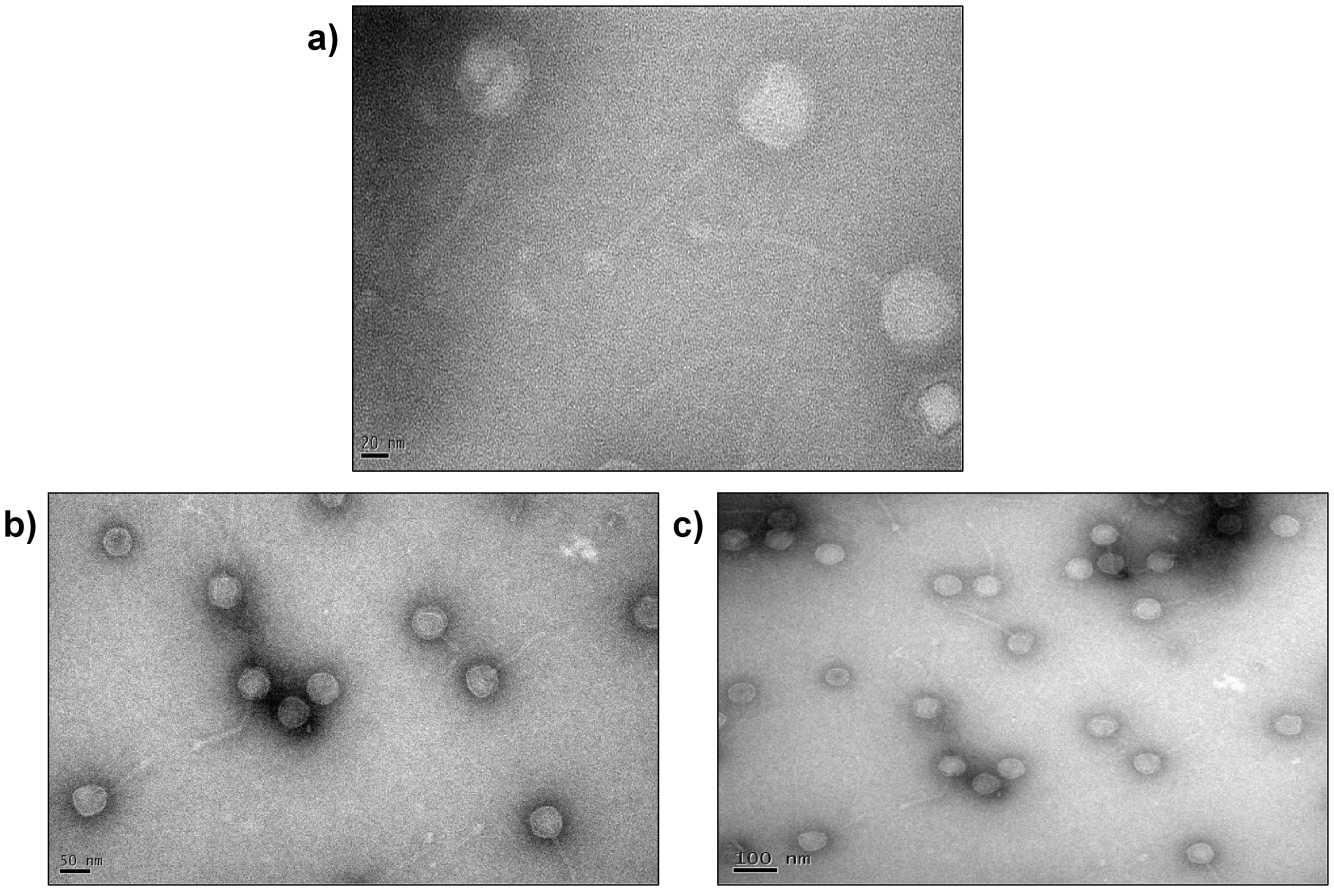
Negatively stained Transmission Electron Micrographs of φIND772PVL.

Our collection of *S. aureus* samples from India has 45 PVL-positive ST772 strains of which 39 are disease isolates and the remaining carrier strains. On the basis of a previous publication, we performed several PCR reactions to classify the PVL phages from our ST772 collection into two types describing whether the genetic structure supports an icosahedral or an elongated capsid. These PCR reactions are based on sequences from the following five PVL phages: φ108PVL, φPVL, φSa2958, φSa2MW, φSLT [8]. PCR was also carried out in our isolates for detection of φSa2USA (PVL phage in USA300) signatures, as described in an earlier publication [21]. Our results show that none of our ST772 isolates could be reconciled with any of the above PVL phages (Table 1). On the other hand, 17 out of 19 ST22 (EMRSA-15) isolates were φPVL-like (data not shown). Recently, Zhang et al isolated and sequenced a PVL phage called φ7247PVL from *S. aureus* ST59 carrying a SCC*mec* type V element [9]. By performing PCR reactions, we observed that our ST772 phages were positive for several genes from φ7247PVL (Figure S1). However, regions of similarity were mainly restricted to the structural genes (*cap* and *por*), with variation in the lysogeny (*ant*) region. We then performed transmission electron microscopy of the PVL phage from ST772 (Fig. 1), which appears to have a quasi icosahedral head ∼54 nm in size and an ∼180 nm-long tail. The overall morphology of the virions is typical of the family *Siphoviridae.* Taken together, these results led us to believe that ST772 PVL phages are different from other known PVL-encoding phages, at least in their genetic architecture, if not in their morphology.

**Table 1:** Characterization of PVL phages from ST772 isolates.

ST (No. of isolates)	MRSA/MSSA N/N	Integrase type	Morphology PCR 1/2 Icosahedral/Elongated	Lineage PCR 3/4 Icosahedral/Elongated	Phage type PCR 5, 6, 7 & 8	φSa2USA	Remarks
ST772 (45)	35/2	2	–	–	–	–	UD*
	1/0	2	–	–	φ PVL	–	UD
	1/0	2	–	–	φ 108PVL	–	UD
	1/0	2,3	–	–	–	–	UD
	1/0	2,3	Elongated	–	φ PVL	–	UD
	0/1	2,3	Elongated	–	–	–	UD
	1/0	1,2	–	–	–	–	UD
	0/1	1,2,3	–	–	–	–	UD
	0/1	2,3,7	–	–	–	–	UD

* UD: Undetermined.

Series of 8 PCRs (1–8) were carried out to identify the two morphological types of PVL- carrying prophages as described by Ma. et al. [8] PCR1- primers specific for icosahedral portal and tail gene, 2- elongated portal and tail gene, 3- Lineage of *lukS-PV* with the tail gene of icosahedral type, 4- Lineage of *lukS-PV* with the tail gene of elongated type, 5, 6, 7, 8 - Phage type specific PCRs for φ108PVL and φ PVL, φ Sa2958, φSa2MW and φSLT respectively. Phage type specific PCR for φSa2USA was carried out as described by Boakes et al [21].

### Genome Sequencing of Clinical Isolates of *S. aureus* ST772

Towards understanding the genetic characteristics of *S. aureus* ST772, we sequenced the genomic DNA of five isolates from different clinical settings: a carrier, and patients with pyomyositis, endophthalmitis, cerebral abscess and pneumonitis (with ID # 60, 118, 333, 120 and 3989) respectively, all typed previously as ST772 [16], [18]. The raw reads obtained from an Illumina HiSeq-1000 sequencer were assembled into contigs using VELVET, and gene predictions made using GLIMMER. The total size of the assemblies and the number of ORFs predicted are consistent with numbers expected from *S. aureus* (Table S1). Mapping of raw sequence reads back to the assemblies estimated insert size distributions that are comparable to that obtained for a control sequencing run, in which reads obtained from USA300 were mapped back to its fully sequenced reference genome (Figure S2). Preliminary comparative analysis of these genomes, in the context of other fully sequenced *S. aureus* genomes, show that the various ST772 genomes form a tight clade, but with each genome carrying a few unique genes (data not shown). Full comparative genomics analysis of these isolates will be presented elsewhere.

### Comparative Analysis of the Sequence of the PVL-carrying Phage in *S. aureus* ST772

To elucidate the genetic characteristics of the PVL-carrying phage in *S. aureus* ST772 (φIND772PVL), we used previously published flanking site information to extract its complete sequence from our contigs [21]. These data are available at http://www.bugbears.in/staph_772_pvl and in Table S2. The 29-bp core sequences located at both ends matched with those in φ7247PVL with 90% sequence identity. The 25-bp sequences of the *attB* site *(attB-L* and *attB-R)* were identified at both ends of φIND772PVL (Table S2). The 25-bp phage attachment sites *attP-L* and *attP-R* had 92% and 100% sequence identity respectively with φ7247PVL. In each case, the entire ∼43 kb PVL phage was contained within a single contig. G+C content of the whole prophage sequence is 33.4%, which is comparable to that of the whole *S. aureus* genome.

We identified 66 ORFs (Table S2) within the φIND772PVL prophage, which could be organized into five contiguous modules, in a manner similar to other PVL phages: (1) lysogeny module, (2) DNA replication/transcriptional regulation module, (3) Structural (packaging, head, tail) module (4) lysis module (5) toxin element (*lukS*-PV and *lukF*-PV). The lysogeny module consists of the integrase gene, which mediates site-specific integration of the phage genome into the bacterial chromosome. The lysogeny control region encodes genes for the cI and cro-like repressors as well as the anti-repressor. Adjacent to these are two genes of unknown function but with strong similarity to hypothetical genes predicted in the genome of *Staphylococcus haemolyticus.* In the replication module, we identified a transcriptional regulator, a single-stranded DNA binding protein, a few other phage replication proteins and a dUTPase, among several proteins of unknown function. The structural gene set included genes encoding the phage terminase major subunit, the S14 family endopeptidase ClpP, the phage major capsid protein, phi13 family major tail protein, the phage tail tape measure protein and phage minor structural protein, among several other hypothetical proteins. The lysis module encodes genes for holin and amidase (endolysin), which contribute to the final step of the bacteriophage lytic life cycle. Finally, the φIND772PVL encodes both PVL toxin components, LukS-PV and LukF-PV.

**Figure 2:**
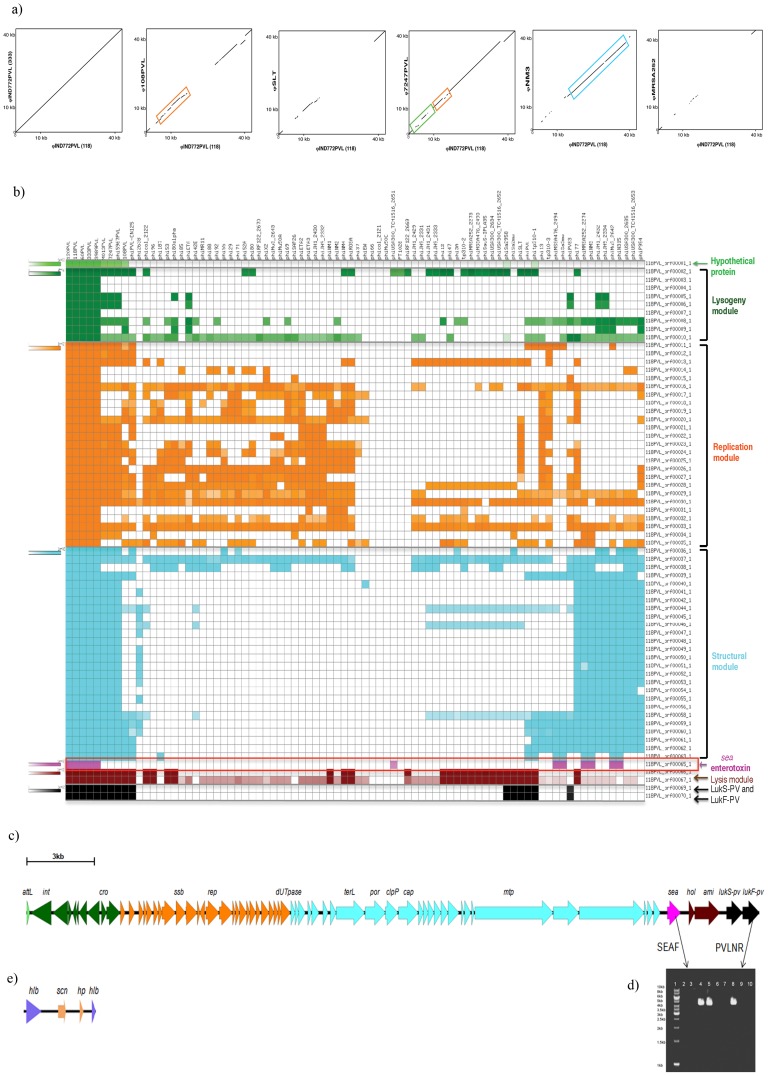
Genomic characterization of φIND772PVL and comparison with other *S. aureus* phages. (a) Dot-plot comparison of φIND772PVL (from strain 118) with each of the following phages: φIND772PVL (from strain 333), φ108PVL, φSLT, φ7247PVL, φNM3 and φMRSA252. The lines (drawn using EMBOSS with a window size of 230 and threshold of 100) on the plot represent similarity between any two phages being compared. Each color represents a module, as indicated in the figure. Various comparisons discussed in the main text are highlighted as follows: region of similarity in the replication module between φIND772PVL and φ108PVL; divergence between φIND772PVL and φ7247PVL in the lysogeny and the replication modules; similarity between φIND772PVL and the non-PVL-carrying φNM3 in the structural module.” (b) Heat-map representing φIND772PVL (from strain 118) ORFs (represented on the vertical axis) and occurrence of their corresponding orthologs in other Staphylococcal PVL and non-PVL phages (represented on the horizontal axis).Genes are color coded as per the phage module they belong (marked in the figure). Intensity of the color is proportional to percent sequence similarity between any two orthologs as determined by Needleman-Wunsch global alignment. The *sea* enterotoxin gene has been highlighted by a red box. Note: The range of color intensities represented in the above heat-map is not very distinct due to high sequence similarity between most of the orthologs. (c) Genomic map of φIND772PVL. Primers used for linkage of *sea* and *lukF-PV* are indicated. Color coding for b and c: green represents a hypothetical protein upstream of the phage integrase marking the beginning of the lysogeny module, dark green: lysogeny module, orange: replication module, cyan: structural module, pink: enterotoxin (*sea*), reddish brown: lysis module and black: *lukS-PV* and *lukF–PV.* (d) Agarose gel picture of *sea-lukF-PV* linkage. PCR for representative strains: Lanes 1∶1 Kb DNA marker, 2: ST8, 3: ST22, 4, 5 and 8: ST772, 6: ST30, 7: ST121, 9: ST1208, 10: ST45. More data are available in SM 7. (e) Genomic map of truncated hlb-converting phage in ST772: Genomic distance between φIND772PVL and hlb-converting phage is about 0.4 Mb.

Dot plot comparisons of the whole sequence of the φIND772PVL with other sequenced PVL phages suggested considerable novelty (Fig. 2a). As suggested by the PCR experiments described above, the φIND772PVL is most similar to PVL phages from *S. aureus* ST59 (φ7247PVL for example), but regions of novelty could be detected even in this comparison.

Comparison of the sequences of the φIND772PVL ORFs to similarly predicted ORF sequences in other *S. aureus* phages show that the PVL-carrying phages are highly divergent from each other and spread across the clustering-based tree (Fig. 2b). However, the φIND772PVL phages cluster with the PVL-carrying phages from ST59 as the two sequence types share many one-to-one bidirectional best-hit orthologs (>90% sequence similarity; Figs. 2b and S3). These similarities are striking in the structural module. Two other PVL-carrying phages (φ108PVL and φPVL-CN125), which cluster alongside the ST772 and ST59 phages, share few orthologs in the structural genes, but have higher similarity in the replication module (Figs. 2b, S3 and S4); however, it must be noted that several non-PVL-carrying *S. aureus* phages also share orthologs of genes from this region of the φIND772PVL sequence (Fig. 2b).

### Presence of the Sea Enterotoxin Gene in the PVL-carrying Phage in *S. aureus* ST772

Compared to all other sequenced PVL phages, including those from ST59, φIND772PVL encodes the following novelty: it carries the *sea* enterotoxin gene at the end of the structural module, before the beginning of the lysis and the toxin modules. A genomic map, showing the linkage between *sea* and the PVL toxin genes, is shown in Fig. 2c. In other *S. aureus* isolates, the *sea* enterotoxin gene is normally carried as part of the *hlb*-converting (βC-φs) phage such as φNM3, whose structural genes share homology with those in the PVL phage in ST772 and ST59. In φNM3, as in φIND772PVL, the *sea* gene is located adjacent to the structural genes. Thus, a single event might have been responsible for the transfer of the structural genes as well as *sea* to the PVL-carrying phage in ST772. PCR analysis of 45 ST772 and 43 non-ST772 isolates from our collection, using one primer derived from the *sea* locus and another from the PVL toxin module, shows that the *sea* is encoded in the PVL phage in every ST772 isolate, but not in any other isolate (Figs. 2d and S5). This indicates that this genetic structure has probably emerged early and unique to the ST772 lineage among Indian isolates.

### The Nature of the *hlb*-converting Phage in *S. aureus* ST772

*hlb*-converting phages are not known to encode the PVL toxin. Our results suggest that the structural genes of the PVL-carrying phages in ST772 and ST59 might have been acquired from *hlb*-converting phages. Given the geographic separation and the genetic distance between these two sequence types of *S. aureus,* we surmise that a possible recombination event occurred independently in the two lineages. ST59 does not appear to contain any integrated, extant *hlb*-converting prophage, as shown by a study of the genome of *S. aureus* M013. Thus ST59 has an intact *hlb* gene, which carries the insertion site for the *hlb* converting phage. On the other hand, ST772 encodes only a ∼3 kb relic of this prophage, as opposed to ∼40 kb of a typical *hlb*-converting prophage (Fig. 2e). The remnant of the *hlb*-converting phage in *S. aureus* ST772 encodes *scn* (staphylococcal complement inhibitor) and two conserved hypothetical phage proteins. This is unique, as none of the previously known immune evasion clusters of the *hlb*-containing prophage, despite being highly variable, is known to carry only the *scn* gene [26].

### Transcript Levels of *sea* and *lukS-PV* in ST772

**Figure 3:**
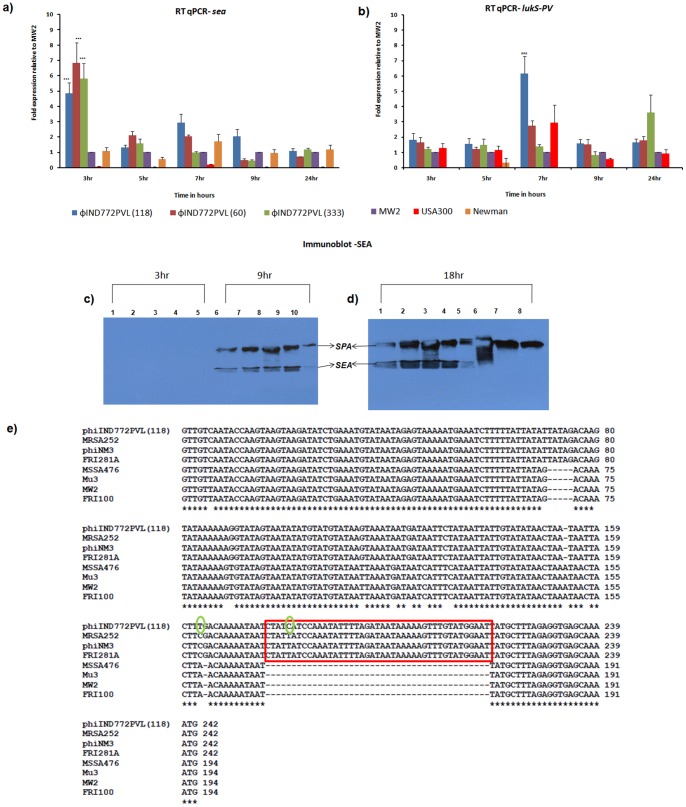
Comparison of expression patterns of *sea* and *lukS-PV* in ST772 and other *S. aureus* isolates. (a) Comparison of transcript levels of *sea* in ST772 (60, 118 and 333), Newman, USA300 and MW2 strains by qPCR. Statistical significant differences were determined by ANOVA and Bonferroni's Multiple Comparison Test. φIND772PVL (118), φIND772PVL (60), φIND772PVL (333), significantly expresses higher amounts of *sea* at 3 hr than the control strain MW2 and Newman p<0.0001. (b) Comparison of transcript levels of *lukS-PV* in ST772 (60, 118, 333), Newman, USA300 and MW2 strains by qPCR. Statistical significant differences were determined by ANOVA and Bonferroni's Multiple Comparison Test. φIND772PVL (118) significantly expresses higher amounts of lukS-PV at 7 hr than the control strain MW2 and USA300 p<0.0001. Color coding for Figure 4 (a) and (b): φIND772PVL (118): blue; φIND772PVL (60): dark red; φIND772PVL (333): olive green; MW2: purple; Newman: orange; USA300: red. (c) Immunoblot of SEA (3 hr and 9 hr). Lanes 1–5 3hr: 118, 60, 333, MW2 and Newman respectively, 6–10 9hr: 118, 60, 333, MW2 and Newman respectively. (d) Immunoblot of SEA (18 hr). Lanes 1∶118, 2∶60, 3∶333, 4: MW2, 5: Newman, 6: USA300, 7∶118Δ φIND772PVL, 8∶118Δ φIND772PVL. (e): Multiple sequence alignment of *sea* promoters *sea1* and *sea2* found in representative *Staphylococcus aureus* strains FRI100 (*sea1)* and FRI281A (*sea2)* reveal that φIND772PVL carries the *sea2* type promoter. The *sea2* type promoter shows an insertion of 43 bp as seen from the multiple sequence alignment. Two Base changes in φIND772PVL are highlighted in green.

**Figure 4:**
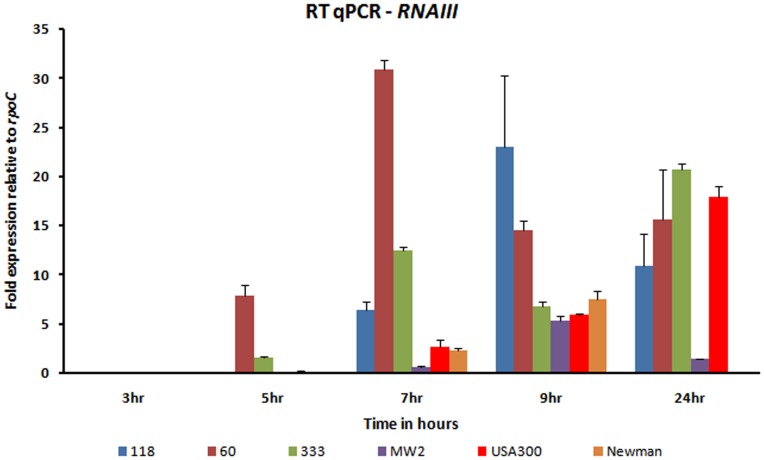
Comparison of transcript levels of *RNAIII* in ST772 (60, 118, 333), Newman, USA300 and MW2 strains by qPCR. Color coding: φIND772PVL (118): blue; φIND772PVL (60): dark red; φIND772PVL (333): olive green; MW2: purple; Newman: orange; USA300: red.

We quantified the transcript levels of both *sea* and *lukS-PV* in ST772 isolates relative to several other *S. aureus* strains: Newman (carrying only *sea* on a *hlb* converting phage), reported to have an ‘intermediate’ expression level of *sea*; MW2 (carrying *PVL* on a PVL phage and *sea* on a *hlb* phage), with *sea* expression level expected to be higher than in Newman; and USA300 (carrying only *PVL* on a PVL phage, but no *sea* gene). Growth curves of all isolates, showing that there is no difference in growth rates is presented in Figure S6. The expression levels of *sea* and *lukS-PV* in each strain are normalized to that of MW2. In all the three ST772 isolates, *sea* is expressed at significantly higher levels at 3 hr compared to both Newman and MW2 (P<0.0001). sea expression is reduced and differ among the various isolates at later time points. On the other hand, *lukS-PV* appears to be expressed in ST772 (118) at significantly higher levels, when compared to MW2 and USA300, at 7 hr (post exponential phase) (P<0.0001) (Figs. 3a and 3b). To test whether these expression patterns could be caused by the quorum-sensing regulator of virulence, *agr*, we performed RT-qPCR against *RNAIII*, a key effector of the *agr* system, using *rpoC* as an internal control. *RNAIII* levels vary among all isolates at all time points, with the expression level being lowest at 3 hr and increasing after 5 hr (Fig. 4), reflecting the *agr*-dependent expression of PVL and *agr*-independent expression of *sea* [27], [28]. In order to verify translation of *sea* into protein and its secretion into the medium, we tested the expression levels of SEA in supernatants of 3, 9 and 18 hr grown cultures by performing Western blots using a commercially available rabbit SEA antibody and anti rabbit IgG conjugated to horse radish peroxidase. All ST772 isolates produce SEA protein with two of the ST772 s making as much SEA as MW2, whereas Newman had the lowest SEA among all of them (Figs. 3c and 3d). SEA was not detected in the 3 hr supernatant (not yet secreted) while the 9 hr supernatant had detectable levels, and the 18 hr supernatant had substantial levels of SEA.

However, analysis of the promoter sequence of *sea* in various sequenced isolates suggests that ST772 has characteristics of intermediate expression while MW2 that of high expression (Fig. 3e). But ST772 has two changes in the promoter region as indicated in Fig. 3e. It remains to be seen whether the high expression observed in our study is a consequence of the novel neighbourhood of the *sea* gene in the PVL-carrying phage or the two changes in the promoter [27].

### Functional Analyses of Wild Type and Phage-cured ST772 (118)

**Figure 5:**
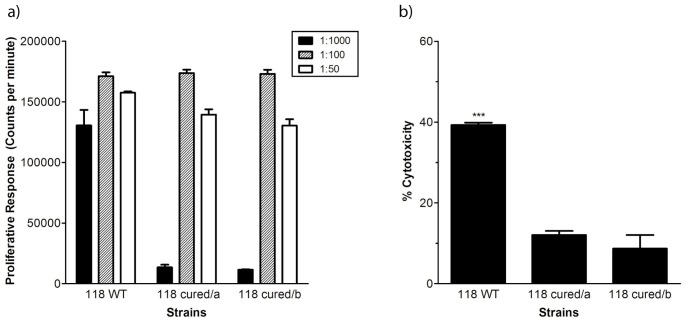
Functional analyses of wild type and phage cured ST772 (118). a) Proliferation assay using human peripheral blood mononuclear cells stimulated with different dilutions of supernatants prepared from overnight cultures of 118 wild type strains and two 118 strains cured of φIND772PVL. Proliferative responses were determined by 3H-thymidine uptake and are presented as mean counts per minute ± SD. The figure shows one representative out of two experiments performed using cells from different donors. b) Cytotoxic activity of 118 wild type strain and two strains cured of φIND772PVL. Primary human neutrophils were exposed to bacterial supernatants for 2 hr after which the cell culture media were analyzed for LDH as detailed in material and methods. The data shows mean± SD from two replicates using cells from three different donors. Statistical significant differences were determined by ANOVA and the Tukey HSD post hoc test. Wild type were significantly more cytotoxic than the two cured strains p<0.0001.

In order to test whether carriage of the *sea* containing PVL-prophage has an impact on bacterial virulence, strain 118 was cured from its prophage by mitomycin C induction. Curing of PVL phages were confirmed by PCR for the absence of *luk-SF PV, sea*, *int, cap* and *por* genes in the cured strain. PFGE patterns of wild type and phage-cured strains were identical except for the presence of one extra large band of roughly 550 kb in the cured phage due to the loss of a *smaI* site inside the PVL phage (picture not shown). Bacterial culture supernatants, containing secreted cytotoxins and superantigens, were prepared and used in functional assays. We assessed the superantigenic activity of the wild type and the cured strains by measuring 3H-thymidine uptake in a proliferation assay using human peripheral blood mononuclear cells (PBMC). The results showed that the wild type strains induced a typical proliferative response with high proliferation evident over a broad concentration range (i.e. dilution range of 1∶50 to 1∶1000) (Fig. 5a). Comparison with two 118 strains that were cured from the prophage revealed a markedly reduced response at the 1∶1000 dilution of the culture supernatant. To assess PVL mediated cell lysis, primary human neutrophils were exposed to bacterial culture supernatants and release of lactate dehydrogenase (LDH) was measured in the cell medium. The two cured strains had significantly reduced cytotoxic activity when compared to the wild type strain (p<0.0001) (Fig. 5b).

Thus, a 118 strain lacking φIND772PVL demonstrated reduced superantigenic and cytotoxic activity, when compared to the otherwise isogenic wild type strain.

## Discussion

Mobile genetic elements (MGE) play a major role in adaptation to stress by transferring genetic material within and between bacterial species. An example is the introduction of SCC*mec* into the Staphylococcal genome after the advent of methicillin [4]. *S. aureus* contains many different types of MGEs including plasmids, bacteriophages, pathogenicity islands, and transposons which carry genes encoding toxins and virulence factors, including the PVL-encoding genes (lukS-PV and lukF-PV) that can be carried by different phages. The role played by this toxin in the virulence of the organism has been most debated and controversial and is still not completely understood [12], [13]. Epidemiologically, PVL has been implicated in deep-seated skin abscesses, furuncles and pneumonia but there are molecular studies contradicting this interpretation [11]. These results are confounded by the high levels of redundancy among putative virulence determinants in *S. aureus*.

We have identified and characterized a novel PVL-carrying phage, φIND772PVL from ST772 *S. aureus.* It is most similar to the PVL carrying phages from *S. aureus* ST59. However, the similarity is limited to the structural genes and to a lesser extent the replication module, with as many as 10 genes (15%) not having any detectable bidirectional best-hit ortholog in any other PVL-carrying phage, including those from the ST59 phages.

Analysis of the sequence of φIND772PVL prophage shows that it is unique in carrying *sea* in its genome. We also show that all ST772 isolates in our collection carry *sea* in the PVL-carrying phage suggesting that this organization developed early in the emergence of the ST772 type in India. This could have emerged as a consequence of a recombination event between a PVL-carrying and a *hlb-*converting phage. *hlb-*converting phages typically carry *sea*, staphylokinase (*sak*), *scn* and chemotaxis inhibitory protein of *S. aureus* (*chp*); all of which are important virulence factors, in their “immune evasion cluster”. However, the content of the immune evasion cluster is variable across strains. The immune evasion cluster in the heavily truncated *hlb-*converting phage in ST772 isolates carries only *scn*, whose individual role in pathogenesis is unclear.

The novel *sea* containing φIND772PVL could play a significant role in bacterial virulence, as it encodes for two major virulence factors: (a) the PVL toxin itself; and (b) a potent superantigen SEA, known to induce strong pro-inflammatory responses contributing to severe systemic manifestations such as septic shock [27], [29], [30]. In ST772, *sea* appears to be highly expressed, despite having a promoter structure that has been previously demonstrated to be characteristic of low and intermediate expression levels [27]. Whether the novel sequence context presented by the PVL carrying phage enables this high expression, remains to be seen. However, the expression of PVL toxin itself has been attributed to two factors: the phage life cycle and the host background [31]. Whether any of these effects, at least those operating at the transcriptional level, are carried over to *sea* in the PVL phage remains an open question.

Our PVL and RNAIII expression data illustrate that their expression levels are different among the three ST772 isolates. This may apply to toxins carried on the core genome as well, as shown by the expression of α-hemolysin levels of the three ST772 isolates (Figure S7). The clinical background of each isolate is different and it is conceivable that amount of PVL and SEA produced are influenced by host as well as bacterial factors like bacterial load, tissue specificity, nature of infection, antibiotics used for therapy, co-morbidities and immune status of the patient [32]. However, it remains to be seen whether and how these factors might have ingrained a gene expression pattern that would also be reflected in *in-vitro* growth conditions as observed here.

Importantly, functional analyses of wild type strains carrying the phage versus strains cured of the phage revealed marked differences in important virulence properties. The wild type strains triggered higher superantigenic responses and were significantly more cytotoxic to neutrophils than the cured strains lacking the prophage. These findings thus demonstrate a potentially important biological role for the φIND772PVL prophage in virulence of *S. aureus* ST772, as it produces high levels of SEA and PVL and thereby, enhanced superantigenic and cytotoxic responses, respectively.

## Materials and Methods

### Ethical Clearance

We have received *S. aureus* isolates from different hospitals in India, and these hospitals have their own ethical boards which give clearance for collection of samples.

Eighty one community associated *S. aureus* isolates from carriers and patients were used in this study which included 57 PVL positive MRSA, and 24 PVL positive MSSA. Molecular characterization of these isolates is described in earlier publications [16], [18]. In addition we also used seven standard *S. aureus* strains and phage DNA (Table S3).

### Identification of *PVL*- encoding Phages

Eight PCRs were performed to determine the genetic relationship between the PVL prophage from ST772 with five other *PVL*-encoding phages (φ108PVL, φPVL, φ Sa2958, φSa2MW, φSLT), using primers and procedures described by Ma et al and Otter et al with slight modifications; similar experiments were performed for φSa2USA as described in Boakes et al [8], [21], [33]. PCRs were also carried out to identify similarities between ST772 PVL phages and φ7247PVL - a newly reported PVL phage from ST59 - by designing primers from its sequence (Table S4). Integrases present in the isolates were determined by a series of PCRs as published earlier [34].

### Induction of Prophages from *S. aureus* and Phage Curing

Overnight grown culture of *S. aureus* (lysogen of PVL phage) was inoculated in 3 ml of BHIB (1∶100 dilution) and grown at 37°C to an absorbance of 0.8 at 595 nm and induced with 1 µg/ml of mitomycin C and incubated at 30°C for 3 hr. After centrifugation at 7,000 rpm for 10 minutes, the cell supernatant was sterile filtered and used for infecting RN4220. Overnight grown RN4220 cell pellets were suspended in resuspension buffer (RB) containing 5 mM CaCl_2_, 50 mM Tris pH 7.6 and 0.15 M NaCl. A portion of the serially diluted filtrate was mixed with equal volume of RN4220 and incubated at room temperature for 20 minutes. To this 8 ml of TSB containing 5 mM CaCl_2_ and 0.6% agar was added, mixed and overlayed on TSB agar plate containing 5 mM CaCl_2_. Plates were incubated at 30°C overnight to form plaques and single plaque was propagated on RN4220.

For phage curing, mitomycin C induced cell pellet was washed twice and serially diluted with RB, an aliquot of which was mixed with 8 ml of TSB containing 5 mM CaCl_2_ and 0.6% agar, and overlayed on a TSB agar plate containing 5 mM CaCl_2_ and incubated at 30°C overnight. Colonies were screened by PCR to verify the absence of *luk-SF PV, sea*, *int, cap* and *por* genes in the cured strain.

### Purification of PVL Bacteriophage and DNA Extraction

The bacteriophage particles were precipitated using Poly Ethylene Glycol (PEG) and NaCl, purified by centrifugation through glycerol step gradient method and DNA was extracted using Proteinase K and SDS as mentioned in Sambrook et al [35].

### Sequencing of ST772 Isolates

We obtained genomic DNA from five *S. aureus* isolates belonging to ST772, fragmented it to 300 to 400 bp, and sequenced these fragments (75-mer paired-end reads) on an Illumina HiSeq 1000 sequencer using the manufacturer’s recommended protocols as described before [25]. PVL phage sequences were derived from the whole genome sequence.

### Sequence Analysis

We obtained draft genomic DNA sequences for five ST772 *S. aureus* isolates (60, 118, 333, 120, 3989) along with reference strain *S. aureus* USA300. The raw-reads for each of the five isolates were filtered for quality and more than ∼8 million reads were obtained with at-least 100X coverage for the 2.8 Mb *S. aureus* genome. The obtained raw reads were assembled using Velvet 1.2.03 [36]; the results of the de novo whole genome assembly are described in Table S1. The sequences have been deposited in Genbank and following are the accession numbers for *S. aureus* isolates 118: AJGE00000000, 120: ALWE00000000, 333: ALWF00000000, 60: ALWG00000000, 3989: ALWH00000000. The φIND772PVL phage was identified from the contigs using PVL insertion sites (Proximal end sequence HQ020554, Distal end sequence HQ020531). The ∼42 kb φIND772PVL phage was found in a single contig and was extracted using a PERL script. DNA sequence of all *S. aureus* associated PVL and non-PVL phages were obtained from NCBI nucleotide ENA database www.ebi.ac.uk/genome/phage.html [37], prophage database http://ispc.weizmann.ac.il/prophagedb and ACLAME http://aclame.ulb.ac.be [38]. ORF prediction was done for all the phages using GLIMMER 3.02 [39]. The phage gene sequences were obtained based on Glimmer ORF predictions. The phage protein sequences were derived from the above gene sequences using EMBOSS transeq [40]. A Bi-directional Best Hit (BBH) phmmer was performed with an e-value threshold of 10^−20^ to define orthologs (hmmer.org version 3.0). Needleman–Wunsch global alignment algorithm was used to determine sequence similarity between the orthologs. The matrix of ortholog occurrences thus obtained was used to cluster the phage sequences, based on Euclidean distances. The hierarchical clustering was performed using Cluster 3.0 [41] and the clusters visualized using TreeView 1.1.6 [42] and the matrix2png web server.

### Verification of the Presence of *sea* in the ST772 PVL Phage

Primers were designed to check the linkage between the enterotoxin A (*sea*) and the *lukF-PV* genes using the 118 (ST772) genome sequence (Gen Bank accession number- AJGE00000000). The following set of primers were used: *entA*-F (5′GGTTATCAATGTGCGGGTGG 3′); *PVL*-R (5′AACTATCTCTGCCATATGGT 3′).

### Transmission Electron Microscopy

Phage suspension was loaded on 200 mesh formvar carbon coated copper grid (Tedpella Inc) and allowed the cells to adsorb on the grid for 4 minutes, excess liquid was removed by blotting. The sample was stained with 2% Uranyl acetate for 30 seconds and imaged under Transmission Electron Microscope [43] (Tecnai, T12 G2 Biotwin, FEI Co.,) with 120 KeV objective aperture. Images were acquired using 4K×2K Gatan Orius CCD camera.

### Real-time Quantitative PCR

Overnight grown *S. aureus* cultures (60, 118, 333, Newman, USA 300 and MW2) were re-inoculated in TSB (1∶1000) and grown at 37°C under shaking (200 rpm). The samples were collected every hour to record the OD at 595 nm to generate a growth curve from which 3, 5, 7, 9 and 24 hr were chosen for RNA extraction. The bacterial cultures were concentrated or diluted to 1 OD and 400 µl of the cultures were taken for total RNA extraction using the Rneasy Mini kit (Qiagen) according to the manufacturer's instructions. One microgram of each RNA was reverse transcribed using Quantitect Reverse transcription kit (Qiagen). cDNA (20 ng) from each isolate was used for real-time quantitative PCR using the Eco Real Time PCR system (Illumina). *sea, lukS-PV and RNAIII* genes were amplified using primers listed in (Table S4) and *rpoC* gene was amplified as an endogenous control. Five sets of experiments were performed and all the reactions were performed in triplicates. The relative transcriptional levels within distinct experiments were determined by using the 2^−ΔΔ*C*T^ method [44].

### SDS-PAGE and SEA Western Blots

Culture supernatants from 3, 9 and 18 hr grown cultures were concentrated using Amicon Ultra (Millipore, Ireland). Equal amount of protein from each isolate was resolved on 12% acrylamide gels and transferred to nitrocellulose membrane using semidry transfer technique. Membrane was blocked using 5% skim milk powder and 5 mM DEPC in PBS pH 6.0 for 20 minutes at room temperature, washed thrice with PBS pH 7.4 containing 0.1% tween 20 (PBST), followed by overnight incubation at 4°C with 1∶10,000 dilution of rabbit anti SEA antibody (Sigma Aldrich, India) in PBS containing 0.05% tween 20 [45]. Membranes were washed thrice with PBST followed by incubation with 1∶20,000 dilution of Anti rabbit IgG-HRP conjugate (Sigma Aldrich) in PBS containing 0.05% tween 20. Membranes were washed thrice with PBST and developed using Rapid Step ECL Reagent (Calbiochem, USA) and the fluorescence was captured on an X-ray film.

### PBMC Proliferation Assay

Human peripheral blood mononuclear cells (PBMC) were isolated using Lymphoprep (Axis-Shield) density centrifugation. Cells were seeded at 2×10^5^ cells/well in 200 µl of RPMI supplemented with 10% FCS, 10 µM of L-glutamine (Thermo Scientific HyClone) and 5X Penicillin/Streptomycin (Thermo Scientific HyClone) in a 96 well plate. The cells were stimulated with different dilutions of bacterial supernatants prepared from overnight cultures of bacterial isolates and incubated at 37°C for 72 hr. After 72 hr the cells were pulsed with 1 µCi of 3H-thymidine (Perkin-Elmer) per well and incubated at 37°C for 6 hr. Ability to induce proliferation of PBMC was assessed as 3H-thymidine uptake measured in beta-scintillation counter [46].

### Cytotoxic Activity Against Neutrophils

Human neutrophils were isolated from fresh peripheral blood from healthy individuals using Polymorphprep (Axis-Shield PoC AS, Norway) density centrifugation. The remaining pellet was resuspended in complete RPMI containing 5% FCS, 2 mM L-glutamine, 100 U/ml penicillin, 100 g/ml streptomycin, 1 M Hepes (all from Invitrogen), counted and resuspended at a concentration of 1×10^6^ cells/ml. Neutrophils were seeded at 5×10^5^ cells/well in 1 ml of complete RPMI in a 24 well plate. The neutrophils were stimulated for 2 hr with bacterial supernatant diluted 1∶50. Lactate dehydrogenase (LDH) leakage to the culture medium of neutrophils was measured with a CytoTox 96 nonradioactive cytotoxicity assay kit (Promega, Madison, WI) according to the manufacturer’s protocol. Maximum LDH release was determined by lysing the cells with lysis solution for 45 minutes. The absorbance was read at 490 nm using a Microplate Manager 6 reader (Bio-Rad, Hemel Hempstead, U.K.).

## Supporting Information

Figure S1 Agarose gel picture for PCRs to identify φ7247PVL.
(PDF)

Figure S2 Box-plot representation of estimated insert size distribution.
(PDF)

Figure S3 Dot plot analysis.
(PDF)

Figure S4 Representation of sequence similarity (Needleman-Wunsch alignment) between φIND772PVL (from strain 118) phage and φNM3, φIND772PVL (from strain 333), φSLT, φ108PVL, φ7247PVLand φMRSA252.
(PDF)

Figure S5 Agarose gel picture of sea-lukF-PV linkage PCR.
(PDF)

Figure S6 Growth curve.
(PDF)

Figure S7 Transcript levels of hla in ST772 isolates.
(PDF)

Table S1 Summary table of de novo genome assembly.
(PDF)

Table S2 Annotation of the PVL phage from S. aureus ST772 (isolate 118).
(PDF)

Table S3 Strains used in the study.
(PDF)

Table S4 List of primers used in the study. Additional data are available at http://www.bugbears.in/staph_772_pvl.
(PDF)
