# Supplementary material for: Genome Sequencing Unveils a Novel Sea Enterotoxin-Carrying PVL Phage in Staphylococcus aureus ST772 from India
Source: PLoS One. 2013 Mar 27;8(3):e60013. doi: 10.1371/journal.pone.0060013 (PMC3609733; doi:10.1371/journal.pone.0060013)
Supplement: Figure S1 — Agarose gel picture for PCRs to identify φ7247PVL. (PDF) [file pone.0060013.s001.pdf]

Figure S1: Agarose gel picture for PCRs to identify φ7247PVL

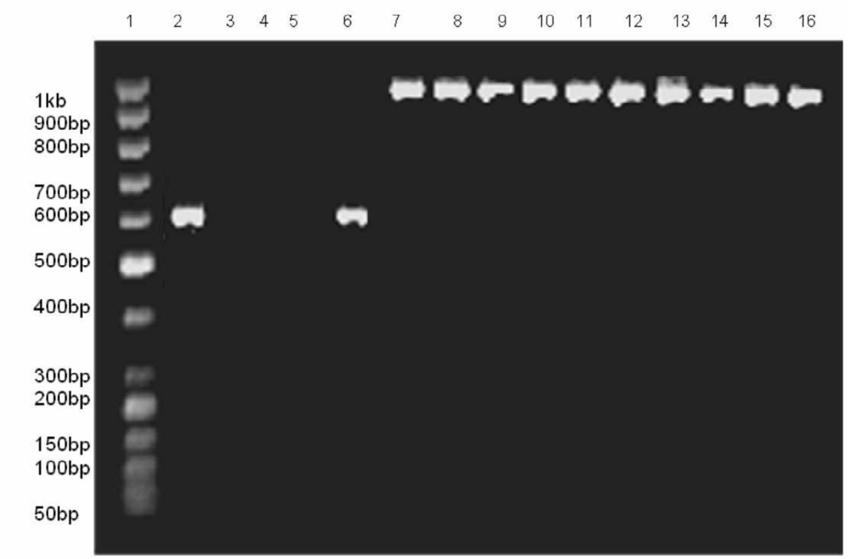

Representative gel picture of *ant, por and cap* gene PCR. Lanes 1: DNA molecular weight marker; 2-6: *ant* PCR (2: N315, 3-6: ST772); 7-11: *por* PCR (7: N315, 8-11: ST772); 12-16: *cap* PCR (12: N315, 14-16: ST772)
