# Supplementary material for: Genome Sequencing Unveils a Novel Sea Enterotoxin-Carrying PVL Phage in Staphylococcus aureus ST772 from India
Source: PLoS One. 2013 Mar 27;8(3):e60013. doi: 10.1371/journal.pone.0060013 (PMC3609733; doi:10.1371/journal.pone.0060013)

Figure S3: Box-plot representation of estimated insert size distribution

Raw reads for each *Staphylococcus aureus* (Sa) isolate were mapped back to the respective contigs using bwa 0.6.2 to determine insert size distribution.

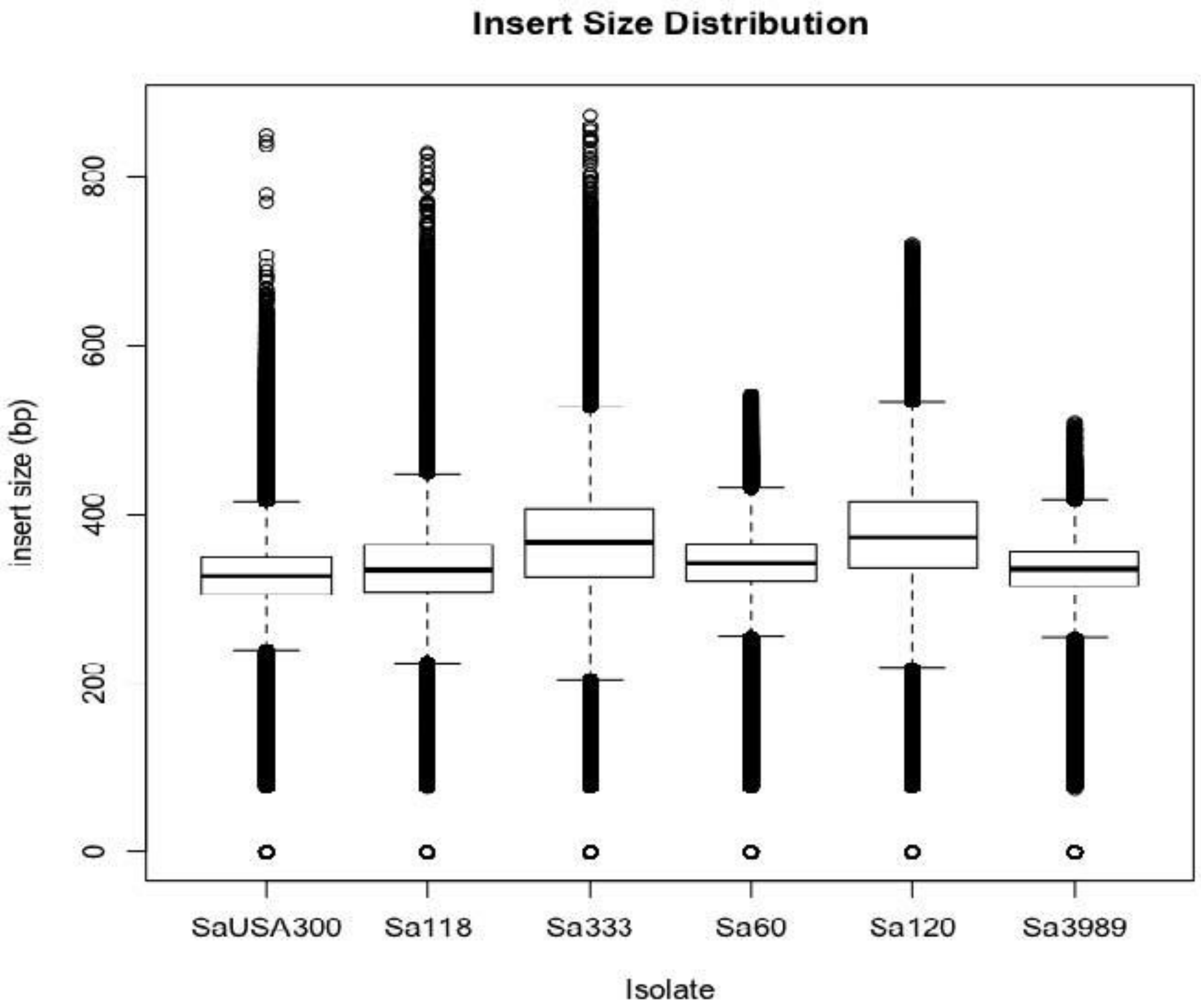

Supplement: Figure S2 — Box-plot representation of estimated insert size distribution. (PDF) [file pone.0060013.s002.pdf]
