# Supplementary material for: Genome Sequencing Unveils a Novel Sea Enterotoxin-Carrying PVL Phage in Staphylococcus aureus ST772 from India
Source: PLoS One. 2013 Mar 27;8(3):e60013. doi: 10.1371/journal.pone.0060013 (PMC3609733; doi:10.1371/journal.pone.0060013)
Supplement: Figure S3 — Dot plot analysis. (PDF) [file pone.0060013.s003.pdf]

Figure S5: Representation of sequence similarity (Needleman-Wunsch alignment) between φIND772PVL (from strain 118) phage and φNM3, φIND772PVL (from strain 333), φSLT, φ108PVL, φ7247PVL and φMRSA252.

Dots on the outlined circle represent ORFs present in each labeled phage. Lines represent corresponding ortholog pairs, while intensity of line color represents percent sequencesimilarity between the two compared ORFs. The *sea* gene orthologs are marked in pink.

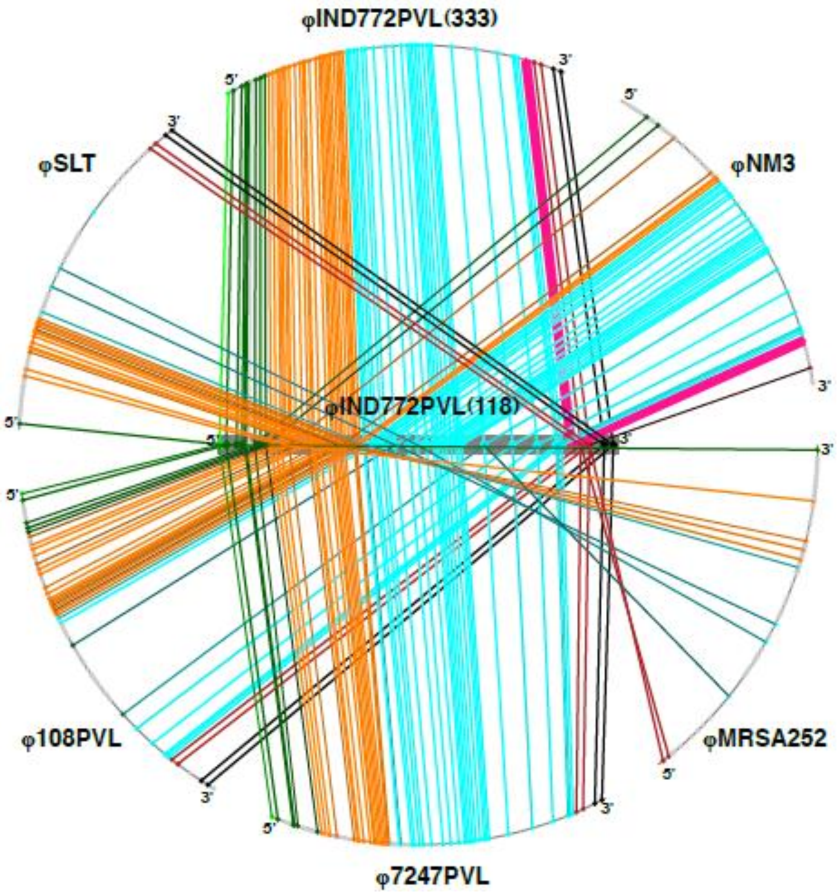

Color coding: green represents a hypothetical protein upstream of the phage integrase marking the beginning of the lysogeny module, dark green: lysogeny module, orange: replication module, cyan: structural module, pink: enterotoxin (*sea*), reddish brown: lysis module and black: *lukS-PV* and *lukF–PV*.
