# Supplementary material for: Genome Sequencing Unveils a Novel Sea Enterotoxin-Carrying PVL Phage in Staphylococcus aureus ST772 from India
Source: PLoS One. 2013 Mar 27;8(3):e60013. doi: 10.1371/journal.pone.0060013 (PMC3609733; doi:10.1371/journal.pone.0060013)
Supplement: Figure S4 — Representation of sequence similarity (Needleman-Wunsch alignment) between φIND772PVL (from strain 118) phage and φNM3, φIND772PVL (from strain 333), φSLT, φ108PVL, φ7247PVLand φMRSA252. (PDF) [file pone.0060013.s004.pdf]

Figure S6: **Dot plot analysis**

118PVL phage was compared with Staphylococcus aureus PVL and non-PVL phages using EMBOSS dotmatcher with windowsize of 230 and threshold of 100 and following dot plots were generated

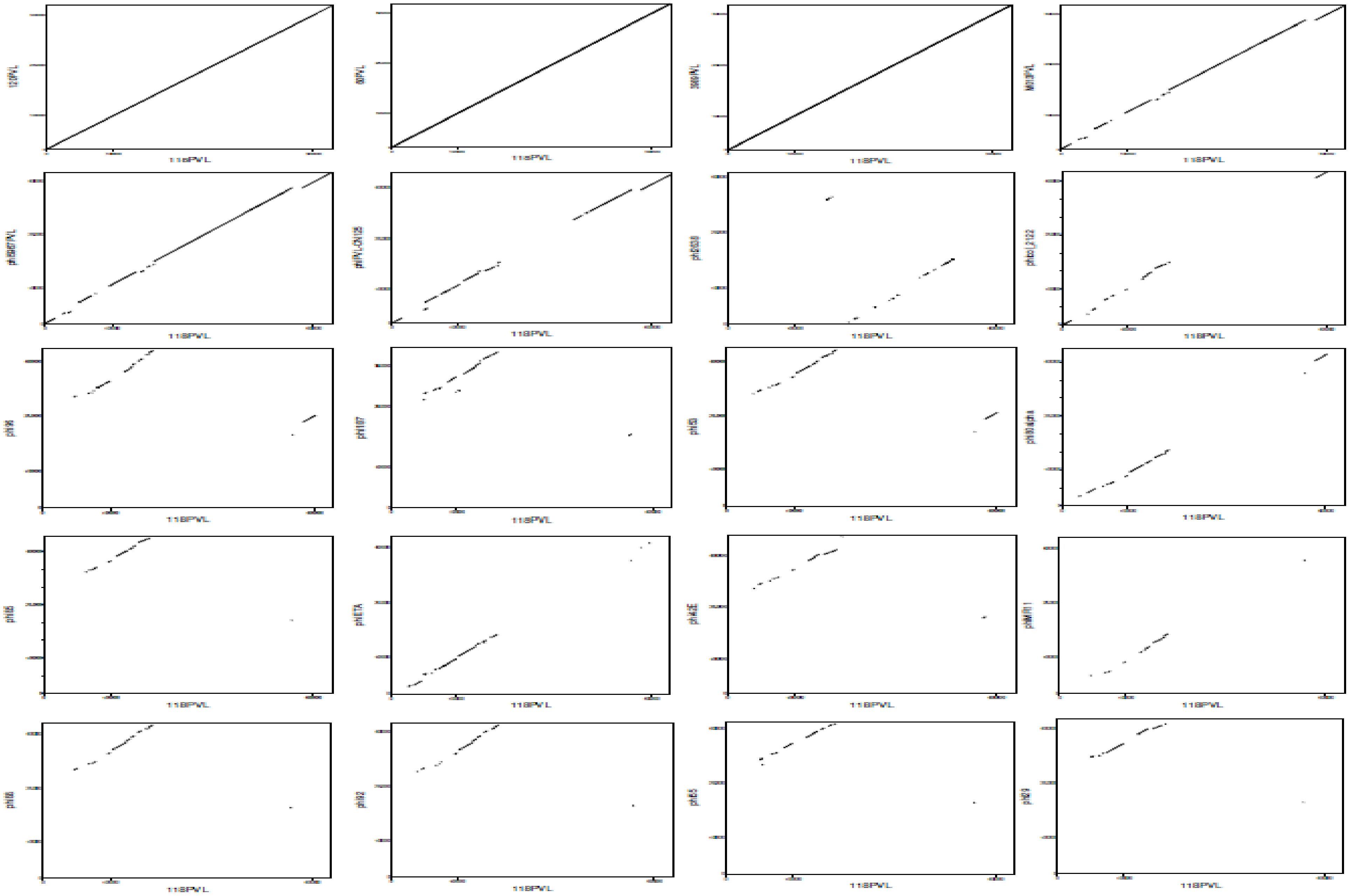

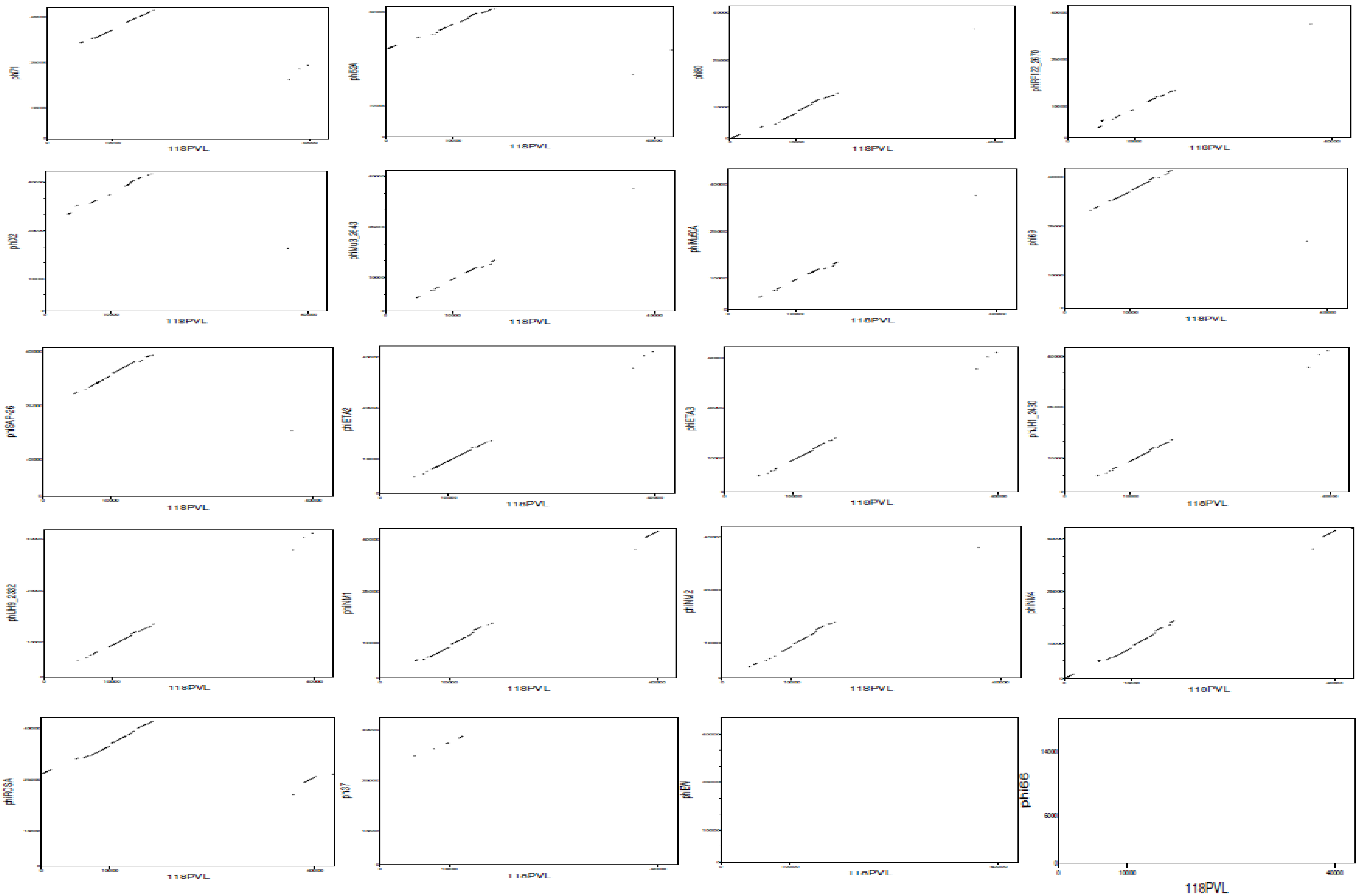
118PVL
phi66
14000
6000
0
10000
40000

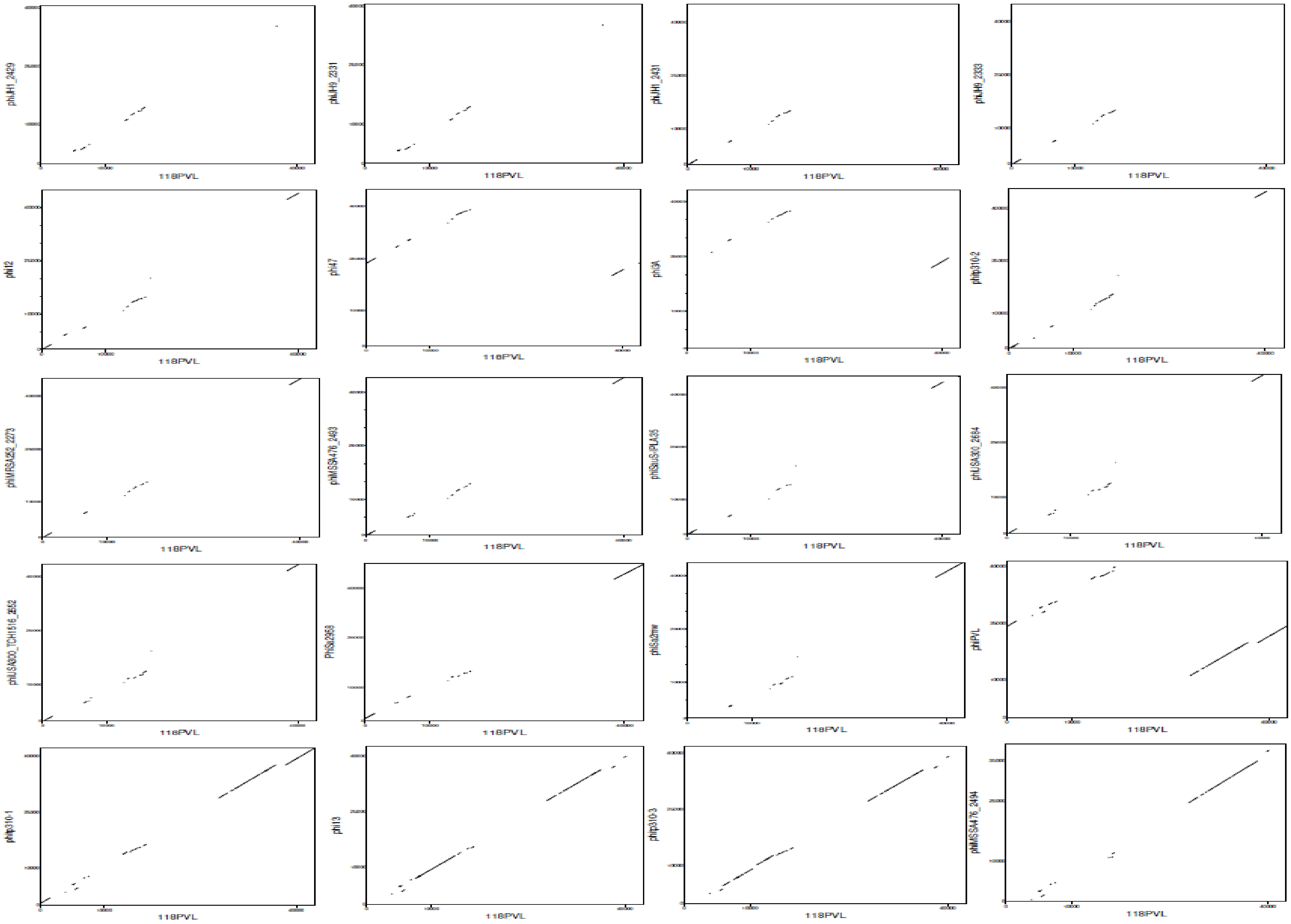
phiJH1_2429
118PVL
phiJH9_2331
118PVL
phiJH1_2431
118PVL
phiJH9_2333
118PVL
phi12
118PVL
phi47
118PVL
phi3A
118PVL
phitp310-2
118PVL
phiMRSA252_2273
118PVL
phiMSSA476_2493
118PVL
phiSauS-IPLA35
118PVL
phiUSA300_2684
118PVL
phiUSA300_TCH1516_2652
118PVL
PhiSa2958
118PVL
phiSa2mw
118PVL
phiPVL
118PVL
phitp310-1
118PVL
phi13
118PVL
phitp310-3
118PVL
phiMSSA476_2494
118PVL

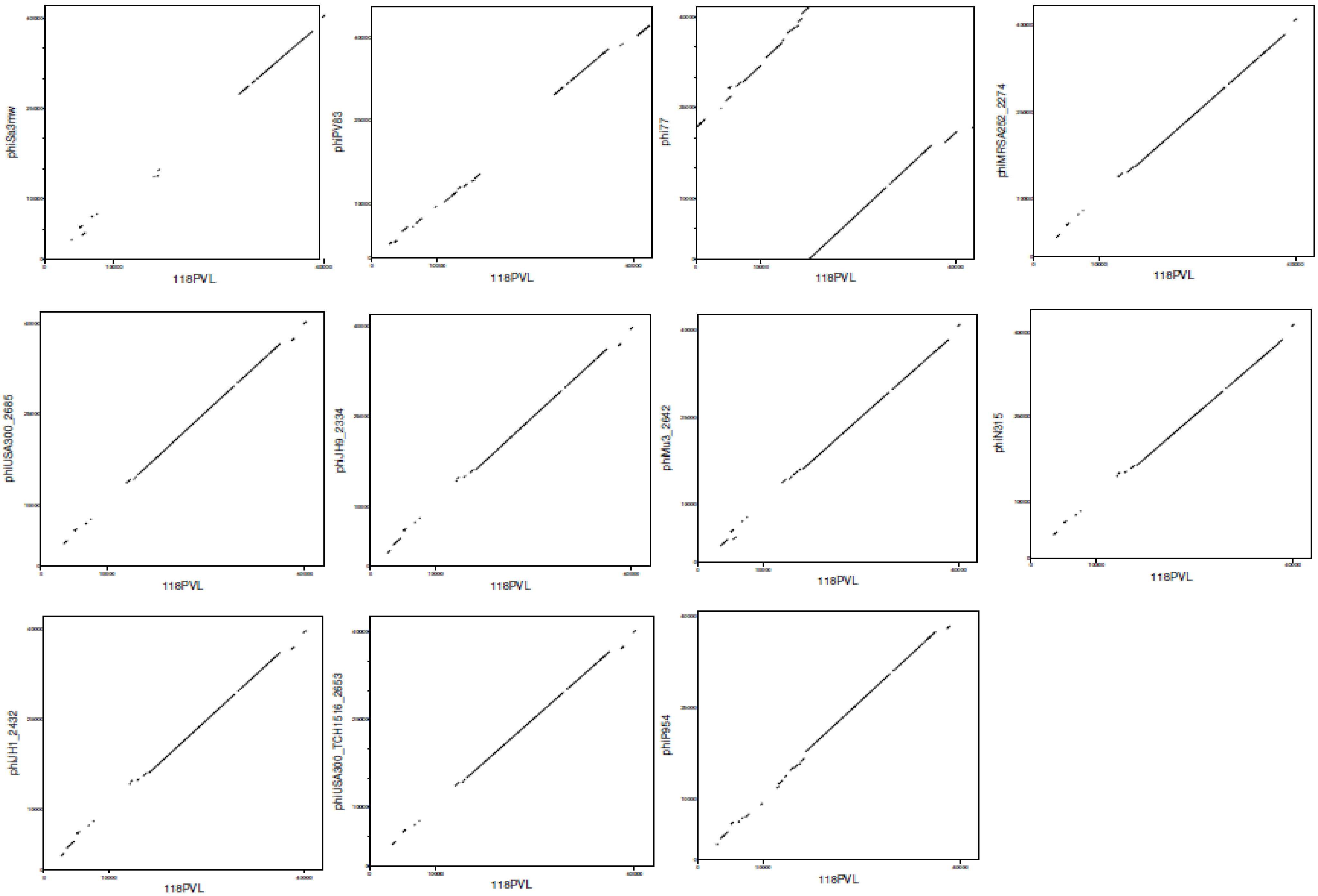

phiSa3mw
118PVL
phiPV83
118PVL
phi77
118PVL
phiMRSA252_2274
118PVL
phiUSA300_2665
118PVL
phiJH9_2334
118PVL
phiMu3_2642
118PVL
phiN315
118PVL
phiJH1_2432
118PVL
phiUSA300_TCH1516_2653
118PVL
phiP954
118PVL
