# Supplementary material for: Genome Sequencing Unveils a Novel Sea Enterotoxin-Carrying PVL Phage in Staphylococcus aureus ST772 from India
Source: PLoS One. 2013 Mar 27;8(3):e60013. doi: 10.1371/journal.pone.0060013 (PMC3609733; doi:10.1371/journal.pone.0060013)
Supplement: Figure S5 — Agarose gel picture of sea-lukF-PV linkage PCR. (PDF) [file pone.0060013.s005.pdf]

**Figure S7: Agarose gel picture of *sea-lukF-PV* linkage PCR**

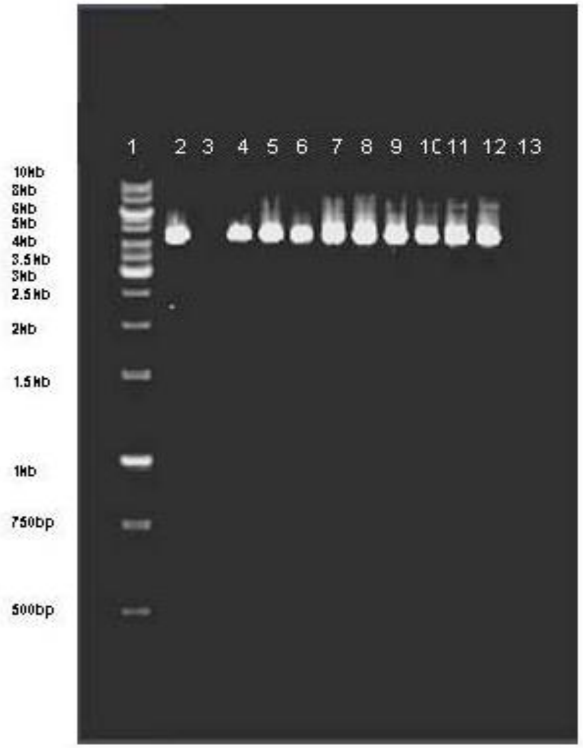


Representative gel picture of *sea-lukF-PV* linkage PCR Lanes 1:1 Kb DNA marker, 2, 4, 5, 6, 7, 8, 9, 10, 11 & 12: ST772, 3: ST5 & 13: ST1
