# Supplementary figures and images for: Genome Sequencing Unveils a Novel Sea Enterotoxin-Carrying PVL Phage in Staphylococcus aureus ST772 from India
Source: PLoS One. 2013 Mar 27;8(3):e60013. doi: 10.1371/journal.pone.0060013 (PMC3609733; doi:10.1371/journal.pone.0060013)

Figure S8: Growth curve

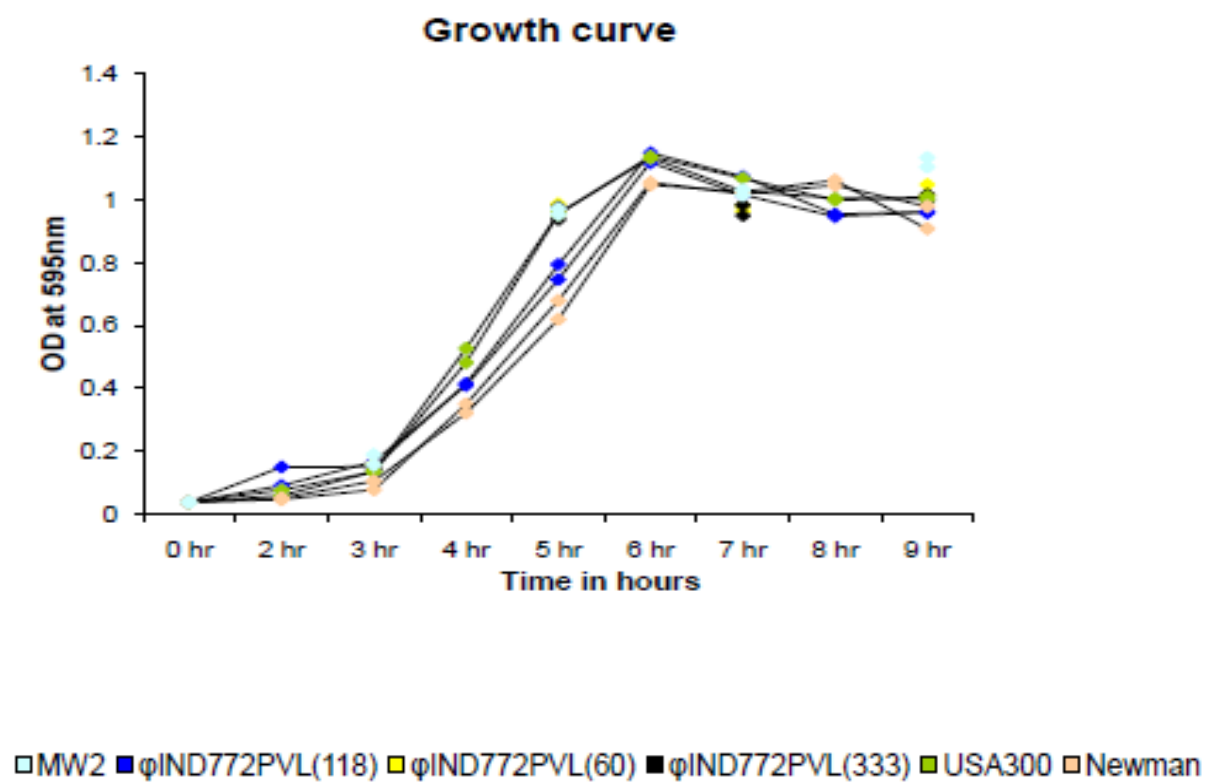

Supplement: Figure S6 — Growth curve. (PDF) [file pone.0060013.s006.pdf]

Figure S9: Transcript levels of *hla* in ST772 isolates

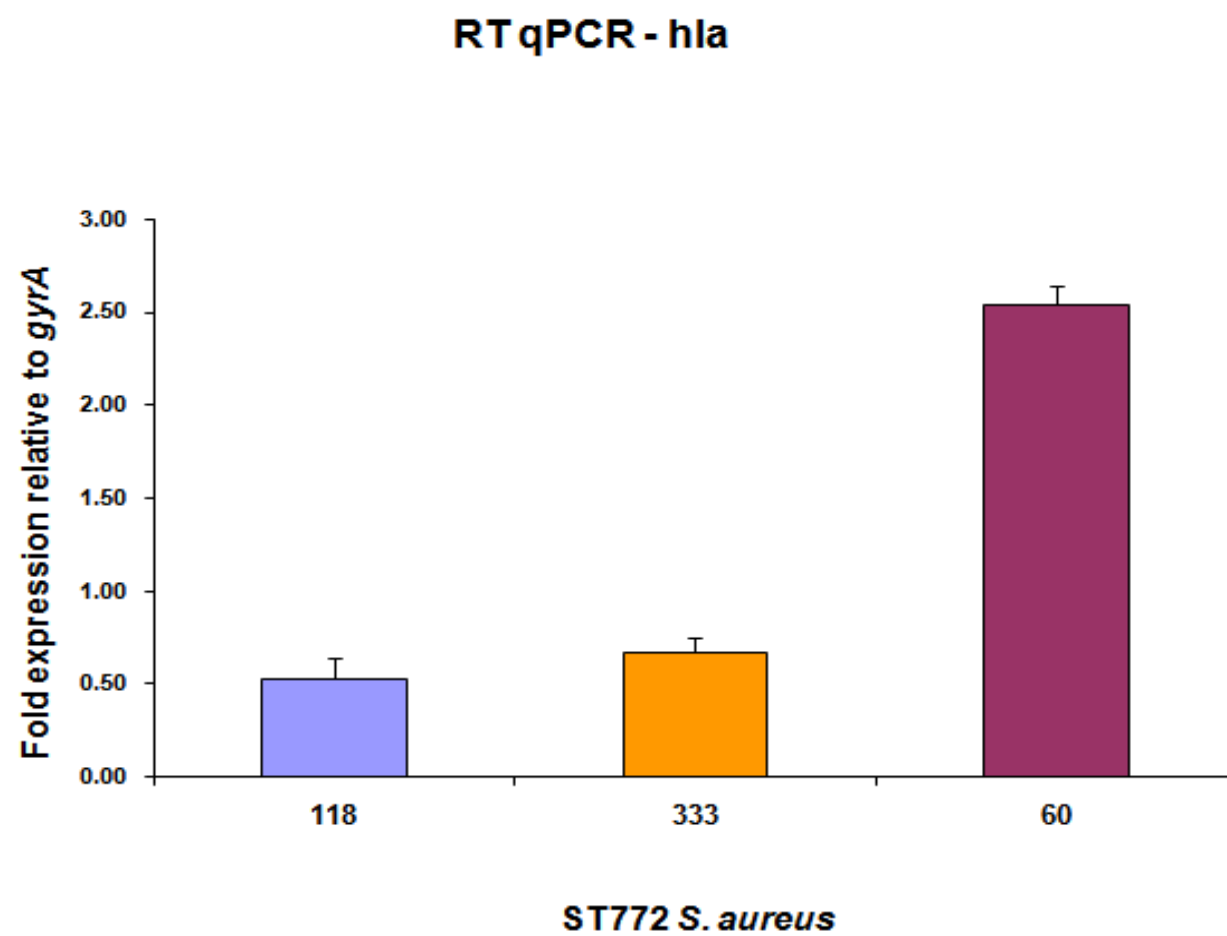

Supplement: Figure S7 — Transcript levels of hla in ST772 isolates. (PDF) [file pone.0060013.s007.pdf]
