# Supplementary material for: Genome Sequencing Unveils a Novel Sea Enterotoxin-Carrying PVL Phage in Staphylococcus aureus ST772 from India
Source: PLoS One. 2013 Mar 27;8(3):e60013. doi: 10.1371/journal.pone.0060013 (PMC3609733; doi:10.1371/journal.pone.0060013)
Supplement: Table S1 — Summary table of de novo genome assembly. (PDF) [file pone.0060013.s008.pdf]

Table S2: Summary table of de novo genome assembly

Isolate	Size of assembly (bp)	Number of Contigs	N50 (bp)	Number of Predicted orfs
**118**	2791839	37	221026	2574
**120**	2782604	45	247775	2605
**60**	2779388	42	216749	2500
**333**	2766547	52	224492	2556
**3989**	2772624	38	298308	2453
**USA300**	2880944	42	287373	2732
