# Supplementary material for: Genome Sequencing Unveils a Novel Sea Enterotoxin-Carrying PVL Phage in Staphylococcus aureus ST772 from India
Source: PLoS One. 2013 Mar 27;8(3):e60013. doi: 10.1371/journal.pone.0060013 (PMC3609733; doi:10.1371/journal.pone.0060013)
Supplement: Table S2 — Annotation of the PVL phage from S. aureus ST772 (isolate 118). (PDF) [file pone.0060013.s009.pdf]

Table S4: Annotation of the PVL phage from *S. aureus* ST772 (isolate 118) φIND772PVL att sites

Number	att site	Sequence start (bp)	Sequence end (bp)	Percent Similarity by Smith Waterman Algorithm
1	attB-L	75	99	96
2	attP-L	129	153	92
3	attB-R	42728	42752	88
4	attP-R	42674	42698	100

φIND772PVL ORFs

118PVL phage			*Homology (% similarity)*[a]				*Homology to others* [b]	*Predicted protein function*
ORF	*Start (bp)*	*End (bp)*	*phi108PVL*	*phi7247PVL*	*phiSLT*	*phiNM3*	*(% Identity)*	
orf01	23	169	41.2	53.6			85% identity against a fragment of a protein from Staphylococcus aureus subsp. aureus IS-M	hypothetical protein
orf02	1417	212	99.3	99.5	99.5			phage integrase
orf03	2376	1495					Staphylococcus haemolyticus (90.1)	hypothetical protein

orf04	2404	2607					Staphylococcus haemolyticus (92.16)	hypothetical protein
orf05	2850	2665		95.2				hypothetical protein
orf06	2992	2837		94.2				hypothetical protein
orf07	3466	3023					Weak hits (20-40% identity) against poorly-annotated gram positive genes	N/A
orf08	4200	3484	76.5	75.3		82.1		cI-like repressor
orf09	4328	4606	87.1					putative cro-like repressor
orf10	4622	5419		53.4		53.4		putative antirepressor
orf11	5432	5692	53.4	100				transcriptional regulator
orf12	5926	6252	100	100				hypothetical protein
orf13	6497	6760	98.9	100	97.7			putative DNA-binding protein
orf14	6772	6939						hypothetical protein
orf15	6940	7260					Staphylococcus prophage phiPV83 proviral DNA (98.75)	hypothetical protein
orf16	7352	7612	98.9	79.3	100	66.7		hypothetical protein
orf17	7622	7843					Staphylococcus phage 69 (100)	hypothetical protein

orf18	7836	8624	63.2				Staphylococcus phage 69 (100)	virulence-related phage protein
orf19	8653	9204	100					Single strand DNA binding protein
orf20	9217	9888	98.7	76.9	77.3			hypothetical protein
orf21	9885	10034	84.4	100	100			hypothetical protein
orf22	10308	10027	98.5	100	100			hypothetical protein
orf23	10374	11144	84.4	100	100			phage replication protein
orf24	11154	11933	98.5	99.6	99.6			phage DnaC-like protein
orf25	11927	12085	98.6	96.2	98.1			conserved hypothetical protein
orf26	12099	12320	88.1	98.6	100			hypothetical protein (annotated as gp21 in S. aureus H19)
orf27	12330	12734	88.1	100				hypothetical protein
orf28	12739	12924	88.7	96.8				hypothetical protein
orf29	12925	13281	68.5	99.2				hypothetical protein (annotated as gp24 in S. aureus WW2703/97)

orf30	13285	13527	92.8	100				hypothetical protein
orf31	13592	14041					S .aureus Newman (100)	conserved hypothetical protein belonging to the YopX superfamily
orf32	14038	14322	53.5				S. aureus D139 (100)	hypothetical protein
orf33	14315	14566	91.7	92.9	89.4	100		hypothetical protein
orf34	14556	14738		85.2		100		hypothetical protein
orf35	14731	15273	81.4		81.4	100		**dUTPase**
orf36	15310	15555		100				Hypothetical Phage protein
orf37	15552	15758	66.7	66.2	64.3	100		hypothetical protein
orf38	15755	16141				99.2		hypothetical protein
orf39	16287	16487	100	100		100		hypothetical protein
orf40	16515	16931		100		100		hypothetical protein, annotated as transcriptional regulator RinA in some cases
orf41	17163	17462		99		100		HNH endonuclease family protein
orf42	17593	17937		100		100		hypothetical protein

orf44	17934	19595	36.5	98.6	45.4	100		phage terminase large subunit
orf45	19611	20798		99.7		99.7		phage portal protein
orf46	20782	21519		99.6	47.8	99.6		S14 family endopeptidase ClpP
orf47	21543	22688		100		100		phage major capsid protein
orf48	22708	22992		100		100		hypothetical protein belonging to the Sec62 superfamily
orf49	22982	23266		100		100		hypothetical protein annotated as gp6-like head-tail connector protein in S. aureus CIG1150
orf50	23250	23612		100		100		hypothetical protein, annotated as head-tail adaptor in S. aureus T0131
orf51	23609	24013		100		100		hypothetical protein, with weak similarities to gp9 from Listeria monocytogenes

orf52	24010	24417		100		100		hypothetical protein, with weak similarities to gp9 from Listeria monocytogenes
orf53	24418	25062		99.5		99.5		phi13 family phage major tail protein
orf54	25188	25328		100		100		hypothetical protein
orf55	25378	25728		100		100		hypothetical protein
orf56	25782	25916		97.8		100		hypothetical protein
orf58	25973	30517	56.6	98.7	34.7	89.8		phage tail tape measure protein
orf59	30514	31998	93.8	90.7		99.8		putative phage tail component in
orf60	32014	35796	99.6	94.9		94.1		phage minor structural protein
orf61	35789	35941	100	84.3		84.3		hypothetical protein
orf62	35987	36274	99	88.5		88.5		hypothetical protein
orf63	36330	36704	98.4	100		100		hypothetical protein
orf65	37125	37898				99.6		enterotoxin type A precursor
orf66	38374	38697	93.5	93.5	93.5			holin
orf67	38708	40162	98.6	97.9	97.7	20.2		amidase
orf69	40552	41490	100	100	100			Panton-Valentine leukocidin chain S precursor

orf70	41492	42469	100	100	100			Panton-Valentine leukocidin chain F precursor

In the above table, the % similarities for the four named strains (labeled as [a]) are based on Needleman-Wunsch global alignments of orthologs identified using bidirectional best-hit phmmer. An entry is made in the homology to others column (labeled as [b]) if none of the other four named strains have an ortholog with >80% sequence identity; the number entered here is based on blastp searches against the NR database. The annotation in the final column is based on the most informative top hit from a blastp search against the NR database.
