# Supplementary material for: Genome Sequencing Unveils a Novel Sea Enterotoxin-Carrying PVL Phage in Staphylococcus aureus ST772 from India
Source: PLoS One. 2013 Mar 27;8(3):e60013. doi: 10.1371/journal.pone.0060013 (PMC3609733; doi:10.1371/journal.pone.0060013)
Supplement: Table S3 — Strains used in the study. (PDF) [file pone.0060013.s010.pdf]

Table S10: Strains used in the study

(A)

**ST type***	**MRSA/MSSA**	**No. of isolates**
ST772	40/5	45
ST22	13/6	19
ST121	0/4	4
ST120	0/3	3
ST30	1/2	3
ST72	1/1	2
ST1208	1/0	1
ST5	0/1	1
ST45	0/1	1
ST199	0/1	1
ST88	0/1	1
Total		81

(B)

**Standard strain**	**ST type**
USA300	ST8
φ108PVL*	CC30
φSa2MW*	ST1
φSa2958*	ST5
φPVL*	-
φSLT*	-
WCH1000	-
Total	7

* PVL- carrying phages

*Molecular characterization of these strains is published elsewhere (16, 18).
