# Supplementary material for: Genome Sequencing Unveils a Novel Sea Enterotoxin-Carrying PVL Phage in Staphylococcus aureus ST772 from India
Source: PLoS One. 2013 Mar 27;8(3):e60013. doi: 10.1371/journal.pone.0060013 (PMC3609733; doi:10.1371/journal.pone.0060013)
Supplement: Table S4 — List of primers used in the study. Additional data are available at http://www.bugbears.in/staph_772_pvl. (PDF) [file pone.0060013.s011.pdf]

Table S11: List of primers used in the study

Primer set 1 was used to amplify anti repressor gene (lysogeny module) and primer sets 2 and 3 were used to amplify portal and capsid genes (structural module) of φ7247PVL. 20ng of the template DNA was amplified at 94°C for 4min, 30 cycles of 94°C for 1min, 55°C for 1min and 72°C for 2mins followed by the final extension for 6mins at 72°C using 0.5 μM of each primer, 250μM of dNTPs and 5 units of Taq DNA polymerase (Fermentas).
Primer set 4 was used to check the linkage between *sea* and *lukF-PV* genes in the PVL phage. 5ng of the template DNA was amplified at 94°C for 4min, 30 cycles of 94°C for 1min, 60°C for 1min and 72°C for 2mins followed by the final extension for 6mins at 72°C using 1μM of each primer, 400μM of dNTPs, 1% of DMSO and 4 units of long PCR enzyme (Fermentas).
RT qPCR was performed using Primer sets 5, 6 and 7. 2ong of the template cDNA was amplified at 95°C for 4min, 40 cycles of 95°C for 30sec, 55°C for 30sec and 72°C for 1min.

Sl .no	Gene name	Primer sequence (5'- 3')	Product size	Description
1	*ant* F	ACTTCATCAAGCATTAGAGGTT	607bp	Identification of φ7247PVL
	ant R	TGTTACTTTTGGCGTCTTACTA		
2	*por* F	AGCTGTCGCTCAAAGTCATT	932bp	
	por R	CAGGATTGTCTGATGGTTCT		
3	*cap* F	TGCAGTAAACAACGGTGAAC	924bp	
	cap R	CCACCAGCTAAATAACCATC		
4	*sea* F	GGTTATCAATGTGCGGGTGG	4621bp	linkage between *sea* and *lukF-PV*
	*PVL*R	AACTATCTCTGCCATATGGT		
5	*sea* F	GGTTATCAATGTGCGGGTGG	102bp	qPCR
	sea R	CGGCACTTTTTTCTCTTCGG		
6	*lukS-PV* F	ATCACATCATTAGGTAAAATGTCTG	244bp	
	lukS-PV R	GTGTTGTTCTTCTCGTCGCATGAGT		
7	*rpoC* F	GACCTACAAAAGACTGGGAATGT	160bp	
	rpoC R	AAATGTGAGAAACTGGAGCAG		
8	*RNAIII* F	GTGATGGAAAATAGTTGATGAGTTGTTT	139bp	
	RNAIII R	GAATTTGTTCACTGTGTCGATAATCC		
